# Natural killer cell as a potential predictive biomarker for early immune checkpoint inhibitor-associated cardiovascular adverse events: a retrospective cohort study

**DOI:** 10.3389/fonc.2025.1556373

**Published:** 2025-07-16

**Authors:** Yujuan Wu, Diansa Gao, Li Tan, Zhulu Chen, Chuan Zhang, Min Mao, Yuxi Zhu, Yue Liu, Zhong Zuo

**Affiliations:** ^1^ Department of Cardiovascular Medicine, Cardiovascular Research Center, The First Affiliated Hospital of Chongqing Medical University, Chongqing, China; ^2^ Department of Gastrointestinal Surgery, The First Affiliated Hospital of Chongqing Medical University, Chongqing, China; ^3^ Department of Oncology, The First Affiliated Hospital of Chongqing Medical University, Chongqing, China; ^4^ Chongqing Clinical Cancer Research Center, Chongqing, China; ^5^ The First Department of General Internal Medicine, Changdu People’s Hospital of Xizang, Xizang, China

**Keywords:** NK cell, immune checkpoint inhibitors, ICI-associated cardiovascular adverse events, biomarkers, immune cell

## Abstract

**Background:**

Peripheral immune cells can predict responses to immune checkpoint inhibitor (ICI) therapy, but their relationship with early ICI-associated cardiovascular adverse events (CVAEs) is unclear. This study aimed to assess the predictive value of peripheral immune cells in early ICI-associated CVAEs.

**Methods:**

Single-cell RNA sequencing (scRNA-seq) dataset from the Gene Expression Omnibus database was used to explore immune cell changes associated with ICI-associated CVAEs. Patients who had received ICI therapy for three cycles at the First Affiliated Hospital of Chongqing Medical University between November 2020 and November 2022 were then included. Patients were stratified into CVAEs and no CVAEs groups and compared peripheral immune cell subsets. Univariate and multivariate regression analyses were conducted to identify CVAEs risk factors. Receiver operating characteristic (ROC) curve analysis determined optimal cutoff values for potential biomarkers. Propensity score matching (PSM) was used to validate the predictive value of baseline NK cell proportion for CAVEs.

**Results:**

ScRNA-seq data revealed decreased CD8+ T and B cell proportions in the CVAEs group, while NK cell proportions increased. Among 203 patients, dynamic changes in the proportion of total T cell, CD8+ T cell, and NK cell differed significantly between groups. Baseline NK cell proportion was identified as an independent risk factor for CVAEs (p=0.009). ROC analysis identified baseline NK cell proportion as a potential predictor of CVAEs (AUC 0.674). The optimal cutoff value was determined to be 16.4%, and this finding was confirmed following PSM.

**Conclusion:**

Baseline NK cell proportion was a potential predictor of early ICI-associated CVAEs.

## 1 Introduction

With the widespread application of immune checkpoint inhibitors (ICIs) in cancer treatment, immune-related adverse events (irAEs) could most commonly occur in the first 3 months of ICI therapy (1). ICI-associated cardiovascular adverse events (CVAEs), although rare, carry a high mortality rate, reaching up to 50% (2). Early prediction and identification of ICI-associated CAVEs were essential for optimizing patient outcomes. However, the effectiveness of biomarkers for early prediction of ICI-associated CAVEs had been suboptimal. Rini et al. showed that elevated baseline levels of cardiac troponin T (cTnT) provided strong predictive value for ICI-associated CAVEs (3). Nevertheless, the predictive capability of cTnT for patients at low risk who received ICI treatment remained uncertain. Delombaerde et al. demonstrated that N-terminal pro-brain natriuretic peptide (NT-proBNP) lacked significant predictive value for the development of ICI-associated CAVEs (4). Additionally, while echocardiography was one of the most precise and widely utilized tools for diagnosing ICI-associated CAVEs, it often failed to detect these events early enough (5). Furthermore, the fact that most patients on ICI therapy did not receive regular echocardiogram monitoring further impaired the effectiveness of early detection and prediction. As a result, there was a notable lack of research addressing the early detection and prediction of ICI-associated CAVEs.

Recent studies have highlighted the significant elevation of clonal cytotoxic Temra CD8+ T cells in the peripheral blood of patients with ICI-associated myocarditis (6). These expanded effector CD8+ T cells exhibit unique transcriptional alterations, suggesting their potential as attractive diagnostic and therapeutic targets (6). Additionally, emerging evidence from preliminary mechanistic studies suggested that central memory CD4+ T cells may play a protective role in ICI-associated myocarditis (7). These findings underscore the crucial role of peripheral blood immune cells in ICI-associated CAVEs. Current researches on ICI-associated CAVEs were limited by small-scale clinical trials, cross-sectional designs, and insufficient focus on early onset, impeding the evaluation of immune cell changes as predictive and diagnostic biomarkers and the understanding of their temporal dynamics. Notably, the changes of immune cell in peripheral blood have been found to be associated with anti-tumor response and can effectively predict the efficacy of immunotherapy (8–10), serving as a biomarker for early diagnosis and predictor of irAEs (11). Therefore, addressing these gaps is essential for advancing our understanding of ICI-associated CAVEs and identifying early predictive biomarkers.

In this study, we used single-cell RNA-sequencing (scRNA-seq) data and conducted a retrospective cohort study involving 203 cancer patients receiving ICIs. Finally, the results underscored the predictive value of baseline NK cell proportion for early ICI-associated CAVEs.

## 2 Methods

### 2.1 Study population

**Figure 1:**
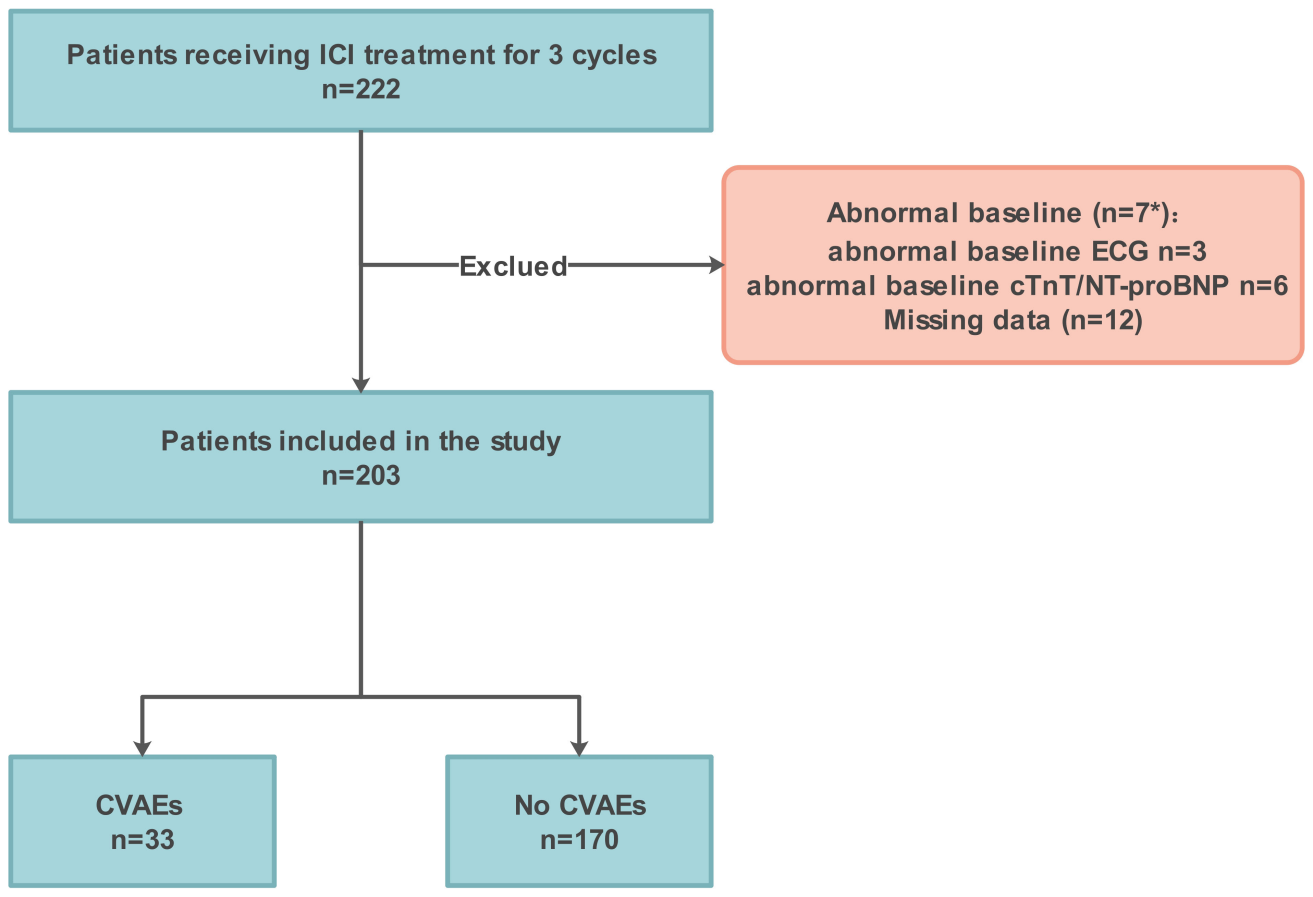
Flowchart illustrating enrolment. ICI, immune checkpoint inhibitor; CVAEs, cardiovascular adverse events. *, The baseline NT-proBNP of the 2 patients with abnormal baseline ECG was also abnormal.

Patients with a pathologic diagnosis of cancer who completed at least 3 cycles of ICI treatment at the First Affiliated Hospital of Chongqing Medical University from November 1, 2020 to November 1, 2022 were enrolled. Patients aged ≥18 years who underwent immunotherapy were included in the study if they had at least one cardiac biomarker (cTnT and NT-proBNP) or ECG after each ICI treatment. The exclusion criteria were as follows: (i) A symptomatic arrhythmia at baseline. (ii) Baseline cardiac biomarkers abnormalities specifically refer to cTnT > 0.030µg/L or NT-proBNP > 300 ng/L. (iii) Patients with heart failure. (iv) Key information was missing, such as lymphocyte subgroups. (**Figure 1**)

### 2.2 Data collection

Data were collected retrospectively from electronic health records including age, sex, body mass index (BMI), smoking, alcohol drinking, past medical history, primary cancer type, ICI type, baseline medications, and cardiac biomarkers (including cTnT and NT-proBNP). The proportion of lymphocytes in participants’ peripheral blood was assessed as a laboratory test during their hospitalization. All examinations were conducted by the Center for Clinical Molecular Medical Detection of the First Affiliated Hospital of Chongqing Medical University using a flow cytometer (BD Canto II and Beckman NAVIOS). The data of lymphocyte counts (including total T cell, CD4+ T cell, CD8+ T cell, CD4+CD8+ T cell, CD4-CD8- T cell, B cell, NK cell, and NKT cell) were also gathered retrospectively from electronic health records. Cardiac biomarkers and lymphocyte counts were performed at baseline and after each ICI treatment.

### 2.3 Flow cytometric cell analysis

Peripheral blood samples were examined by flow cytometer (BD Canto II and Beckman NAVIOS), stained with CD3-FITC/CD16 + 56-PE/CD45-percp-Cy5.5/CD4-PC7/CD19-APC/CD8-APC- Cy7 (BEIJING TONGSHENG SHIDAI BIOTECH; Cat: Z6410010). For absolute counting, 20 μL of premixed antibodies were carefully deposited 0.5 cm above the microbead-free zone in TruCOUNT™ tubes using reverse pipetting technique. Following precise aspiration of 50 μL vortexed whole blood/control material, samples were gently vortexed (3 sec) and incubated protected from light (15 min, RT). Erythrocyte lysis was achieved with 450 μL 1× BD Pharm Lyse™ solution (10 min, RT), followed by immediate acquisition post-lysis verification. In peripheral blood (**Supplementary Figure S1**), total T cell (CD3+ T cell), CD4+ T cell, CD8+ T cell, CD4+CD8+ T cell (CD3+CD4+CD8+ T cell), CD4-CD8- T cell (CD3+CD4-CD8- T cell), B cell (CD19+ B cell), NK cell (CD3-CD16/CD56+ cell), and NKT cell (CD3+CD16/CD56+ cell) within the lymphocyte gate are calculated as a percentage of CD45+ lymphocyte. Gating strategy detailed in **Supplementary Figure S1**.

### 2.4 Outcomes

The outcome of this study was the dynamic changes in immune cells in the peripheral blood during early ICI-associated CVAEs. According to the 2021 American Society of Clinical Oncology (ASCO) Guideline, ICI-associated CAVEs are primarily graded based on cardiac biomarkers and ECG findings (12). As a result, consistent with previous research (13, 14) and the ASCO guideline (12), CVAEs were defined as new ECG abnormalities or abnormal cardiac biomarkers. Among them, abnormal biomarkers were defined as cTnT > 0.030 µg/L or NT-proBNP > 300 ng/L. These cutoffs were selected according to manufacturer-recommended 99th percentile upper reference limits with optimal precision (coefficient of variation <10%). ECG abnormalities were defined as tachyarrhythmias (including atrial fibrillation/flutter and ventricular tachycardia) or bradyarrhythmias (such as sinus bradycardia and second-degree or higher atrioventricular block). Among the 33 cases of CVAEs, 5 cases of myocarditis were diagnosed by a cardiologist.

### 2.5 Quality control and integration of single-cell RNA-seq data

scRNA-seq data (GSE180045) from the Gene Expression Omnibus database were pre-processed using the Seurat v4 R package. In the dataset GSE180045, there are 10 peripheral blood mononuclear cell (PBMC) samples from individuals. The GSE180045 dataset included a total of 10 peripheral blood single-cell sequencing samples, which consist of 3 non-myocarditis irAE samples, 3 no irAE samples, and 4 myocarditis irAE samples. After excluding 3 samples with non-CVAE irAEs, our study included 7 samples: 3 with no irAEs and 4 with ICI-associated myocarditis. Several steps were undertaken to filter out low-quality data. First, cells expressing fewer than 500 or more than 5,000 genes were excluded. Additionally, cells with a mitochondrial gene expression proportion greater than or equal to 20% or a red blood cell gene expression proportion greater than or equal to 5% were removed. Finally, cells containing more than 400 and fewer than 25,000 unique molecular identifiers (UMIs) were retained for subsequent analysis. To integrate and embed individual cells from different samples into a shared low-dimensional space, Seurat’s reciprocal principal component analysis (RPCA) function “IntegrateData” was used to perform batch effect correction and normalization. The resulting integrated matrix was utilized for clustering and cell type classification.

### 2.6 Unsupervised clustering analysis and broad cell type identification

Following the generation of the integrated matrix, unsupervised graph-based clustering was performed using Seurat’s default parameters unless otherwise specified. Briefly, the UMI count matrix was normalized using the “NormalizeData” function with default settings. Variable gene selection was carried out using the “FindVariableFeatures” function, applying the “vst” method. For all cell type clusters, 3000 variable genes were selected. After regressing out the number of UMIs, the dimensionality of the dataset was reduced. Clustering was performed with the “FindClusters” function at 10 PCs and a resolution of 1.0.

The “FindAllMarkers” function was employed to identify unique genes for each cell subpopulation, with the top 20 unique genes used for cell type annotation on the Annotation of Cell Types (ACT) website (http://xteam.xbio.top/ACT/). The following markers were employed for the identification of specific immune cell types: LTB, MAL, AQP3, IL32, CDC14A, and TNFAIP3 for CD4+ T cells; CD8A, CD8B, and CD247 for CD8+ T cells; MS4A1, TCL1A, IGHM, IGHD, IGHA1, IGHA2, IGHG1, IGKC, IGLC3, JCHAIN, CD79A, CD79B, CD19, BLK, and BANK1 for B cells; GNLY, KLRF1, KLRB1, CD7, and KLRD1 for NK cells; and PRF1, CX3CR1, and PTPRC for NKT cells (**Supplementary Table S1**, **Supplementary Figure S2**). After filtering to retain only lymphocytes, t-distributed stochastic neighbor embedding (t-SNE) clustering was conducted on 10 PCs. The proportions of each lymphocyte type across the two groups were visualized using the “ggplot2” package.

### 2.7 Statistical methods

Continuous variables were summarized as mean [SD] or median [interquartile range, IQR], and categorical variables as frequencies and percentages. Longitudinal comparisons of immune cell proportions across multiple treatment cycles were conducted using one-way analysis of variance (ANOVA), with baseline measurements serving as the reference timepoint. The differences in immune cell subsets between patients with and without CVAEs across different treatment cycles were analyzed using the Wilcoxon rank-sum test for p-value. This nonparametric statistical approach was consistently applied to evaluate disparities in total T cell, CD8+ T cell, and NK cell populations when comparing various confounding factors. Univariate logistic analysis was used to analyze risk factors for CVAEs. Variables with P-value < 0.1 and other factors that may influence the outcomes were subsequently considered for multivariate logistic analysis with a stepwise regression method. Odds ratios were calculated for every covariate along with the adjusted P-value. The receiver-operating characteristic (ROC) curve was performed to identify the ability of baseline NK cell proportion in predicting CVAEs. To further validate the predictive value of baseline NK cell proportion for ICI-associated CAVEs, we employed propensity score matching (PSM) to mitigate confounding factors inherent in retrospective studies (15). We identified participants’ demographic characteristics, past medical history, and other baseline modifiable variables as significant confounding factors that could substantially impact the outcomes. In the PSM model, the following confounding factors were included: age, gender, BMI, smoking status, alcohol consumption, coronary artery disease, hypertension, and cardiovascular medications. After calculating the propensity scores, we applied a 1:1 nearest neighbor matching strategy. Following the matching process, we conducted chi-square tests on the matched intervention and control groups to evaluate the effectiveness of the intervention.

A two-sided p value <0.05 was considered significant. Statistical analysis was conducted using SPSS 26.0 statistical software (IBM, Armonk, New York, USA). PSM was based on the “MatchIt” package, and data visualization was performed using “ggplot2” package in R software version v4.2.1.

### 2.8 Ethical approval and informed consent

The study was conducted in accordance with the Declaration of Helsinki (as revised in 2013). This study was approved by the Ethics Committee of The First Affiliated Hospital of Chongqing Medical University (ethics number, 2022-31). The Ethics Committee reviewed and determined that informed consent was waived due to the retrospective, anonymous, and observational nature of the study.

### 2.9 Patient and public involvement

Patients and the public were not involved in the design, or conduct, or reporting, or dissemination plans of the research.

## 3 Results

### 3.1 ScRNA-seq profiling of immune cell subsets populations in ICI-associated CVAEs

**Figure 2:**
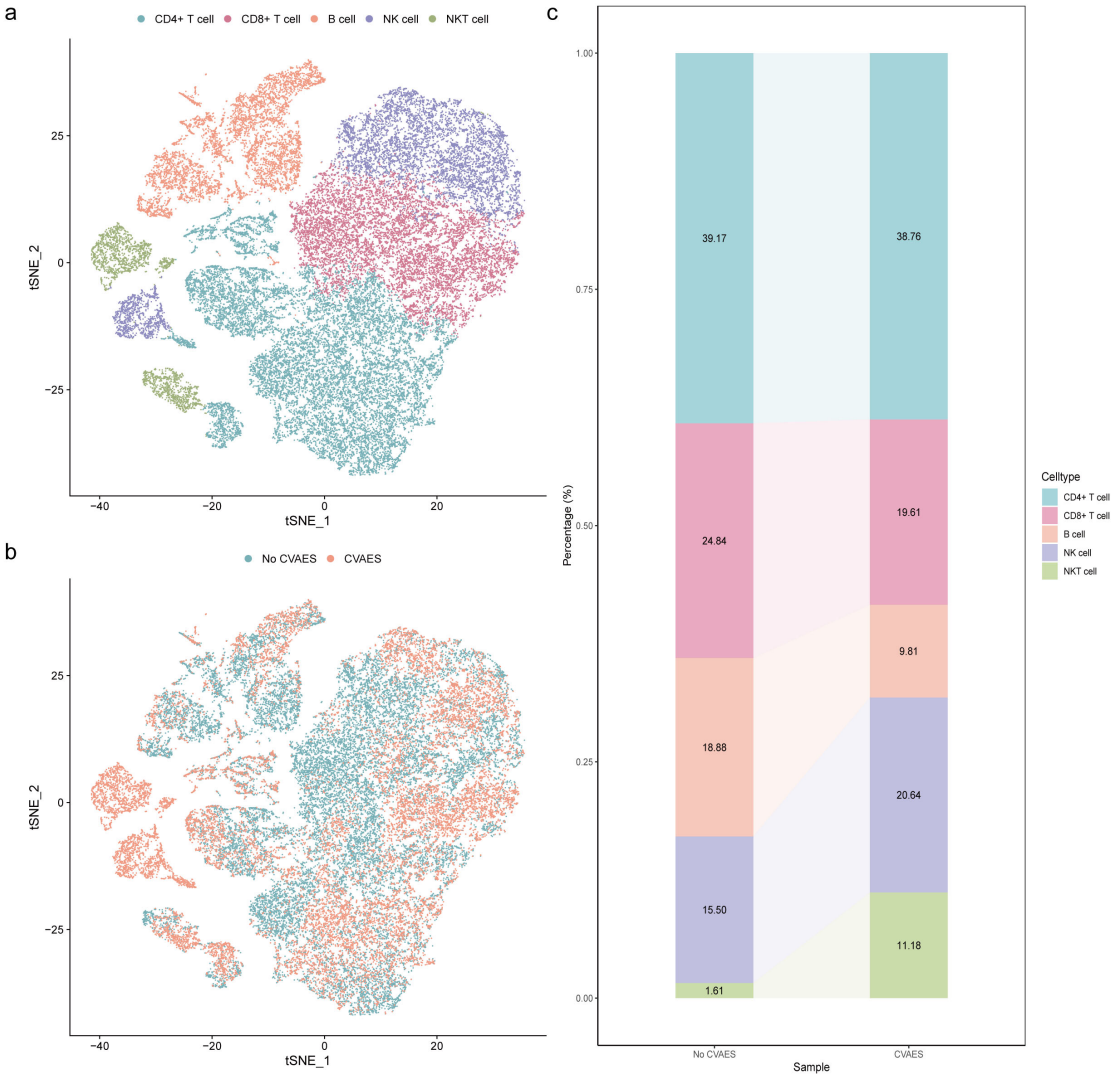
Single-cell atlas of immune cell subsets in peripheral blood. **(a)** The t‐SNE plot identified 5 cell types in peripheral blood. **(b)** The t‐SNE plots of cells clustered in different groups. **(c)** Average percentage of assigned cell types in different groups. CVAEs: cardiovascular adverse events.

To investigate the changes in peripheral blood immune cells during ICI-associated CVAEs, we utilized the scRNA-seq dataset from Zhu et al., which included 4 patients with ICI-associated CVAEs and 3 healthy control subjects (6). After quality control and filtering, a total of 164512 cells were aggregated and visualized using graph-based t-SNE. Five major cell populations (CD4+ T cell, CD8+ T cell, B cell, NK cell, and NKT cell) were identified based on the cell type annotation on the ACT website with the top 20 unique genes for each cell subpopulation (**Supplementary Table S1**). The relative abundance of the five primary cell populations exhibited differential trends between patients with and without CVAEs. Specifically, we observed a reduced proportion of CD8+ T cell and B cell in the CVAEs group, whereas the proportion of NK cell was increased (**Figure 2**).

### 3.2 Patient characteristics

To further elucidate the role of peripheral blood immune cells in ICI-associated CVAEs, we conducted a retrospective cohort study. 203 were included in the final analysis, after 19 patients excluded due to abnormal baseline data (n=7) and missing data (n=12) (**Figure 1**). The baseline clinical data of the patients are shown in **Table 1**. The median age was 62 (51-73) years, with 79.80% being male. A total of 203 cancer patients treated with ICI were diagnosed with lung cancer (62.07%), esophageal cancer (33.50%), and others (4.43%). Among them, 19 patients (9.36%) were prescribed cardiovascular medications. All patients received one type of ICIs regimens, with sixteen patients (7.88%) receiving ICI monotherapy. Thirty-three patients (16.26%) developed CVAEs without chest pain, amaurosis, syncope, or edema.

**Table 1:** Baseline characteristics in all patients. Normal distributed variables are presented as Mean [SD], abnormal distributed variables as median [IQR], nominal variables as number and percentage.

Characteristics			N=203	%
Age (years)		Median [IQR]	62 (11)	
Gender		Male	162	79.803
BMI (kg/m2)		Mean [SD]	22.74 (2.82)	
Smoking			102	50.246
Alcohol drinking (n, %)			63	31.034
Past medical history (n, %)				
Coronary artery disease			9	4.433
Hypertension			28	13.793
Primary cancer type (n, %)				
Lung cancer			126	66.010
Esophageal cancer			68	35.468
Other cancers			9	4.433
Cancer treatment (n, %)				
ICI monotherapy			16	7.882
Chemoimmunotherapy			187	92.118
ICI types (n, %)				
Camrelizumab			143	70.443
Pembrolizumab			8	3.941
Tislelizumab			34	16.749
Cindilimab			9	4.433
Toripalimab			2	0.985
Durvalumab			4	1.970
Atezolizumab			1	0.493
Nivolumab			2	0.985
Vital Signs				
Heart Rate (bpm)		Median [IQR]	80.00 (11.00)	
Admission SBP (mmHg)		Mean [SD]	123.69 (17.43)	
Admission DBP (mmHg)		Mean [SD]	75.95 (11.30)	
Cardiovascular drugs (n, %)			19	9.360
ACEI/ARB/ARNI			6	2.956
Beta-Blocker			4	1.970
CCB			9	4.433
Cardiac Biomarkers				
cTnT (µg/L)		Median [IQR]	0.008 (0.003)	
NT-proBNP (ng/L)		Median [IQR]	55 (59)	
Outcomes				
CVAEs	Myocarditis		5	2.463
	Other CVAEs		28	13.793
no CVAEs			170	83.744

BMI, body mass index; ICI, immune checkpoint inhibitor; ACEI, angiotensin converting enzyme – inhibitor; ARB, angiotensin receptor blocker; ARNI, angiotensin receptor-neprilysin inhibitor; CCB, calcium channel blocker; CVAEs, cardiovascular adverse events.

### 3.3 Changes in immune cell subsets proportions during ICI treatment

**Figure 3:**
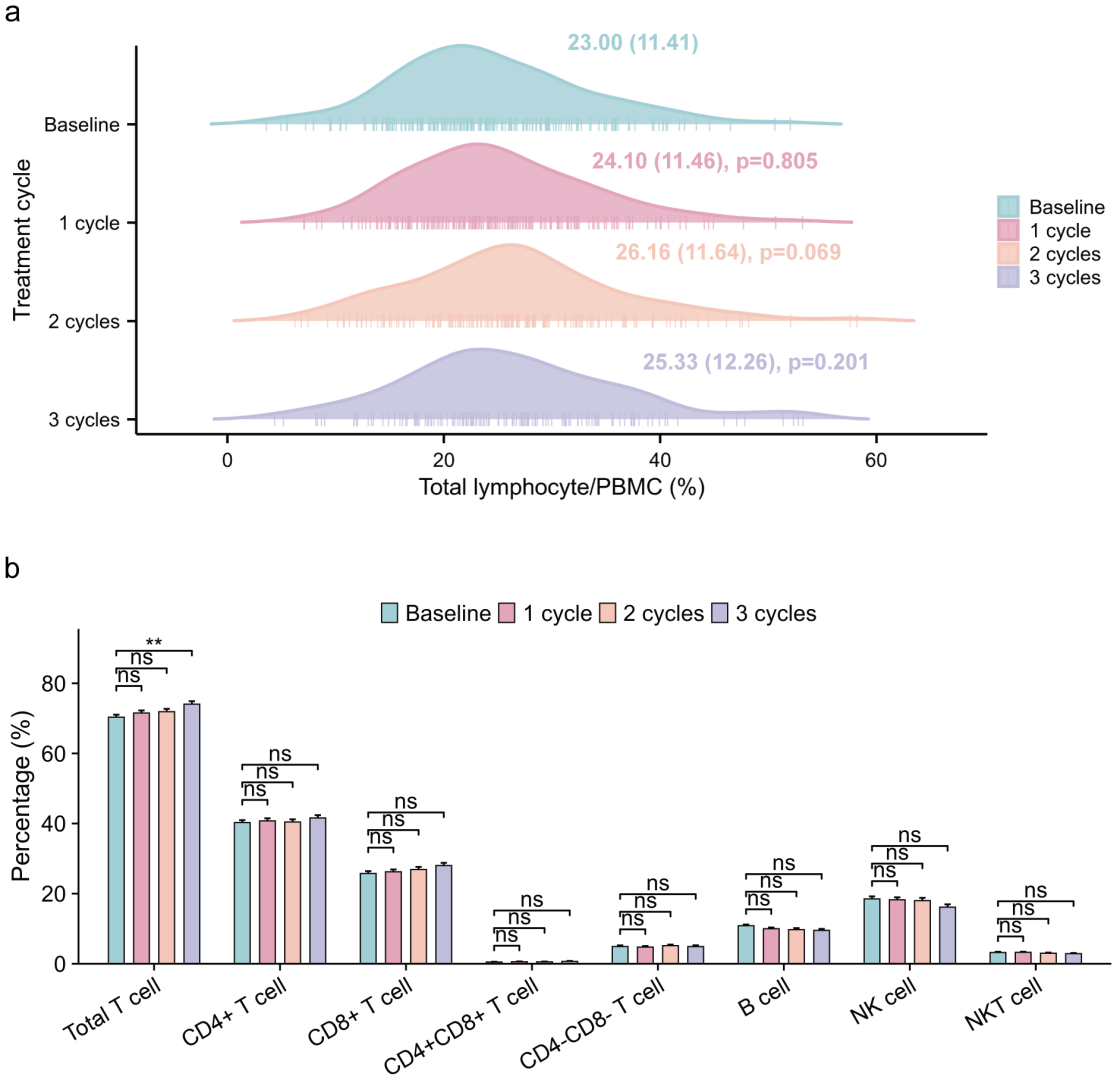
Changes in lymphocytes and immune cell subsets proportions of all patients during ICI treatment. **(a)** longitudinal variations in total lymphocyte/PBMC (%) (median [IQR]) across baseline and three treatment cycles, with one-way ANOVA-derived p-values quantifying statistical differences relative to baseline measurements. **(b)** longitudinal variations in immune cell subsets proportions/lymphocyte(%), employing identical statistical methodology for inter-cycle comparisons. ICI, immune checkpoint inhibitor; PBMC, peripheral blood mononuclear cell. ns p: >0.05; **p: <0.01.

We analyzed the flow cytometry data of peripheral blood samples to examine the changes in immune cell subsets proportions during ICI treatment. During the course of immunotherapy, the proportion of lymphocyte in all patients remained relatively stable compared to baseline (**Figure 3a**). Although no significant differences in lymphocyte subsets were observed between the baseline and other treatment cycles, a gradual increase in the proportions of total T cell, CD4+ T cell, and CD8+ T cell was observed, accompanied by a corresponding decrease in the proportions of B cell and NK cell (**Figure 3b**).

**Figure 4:**
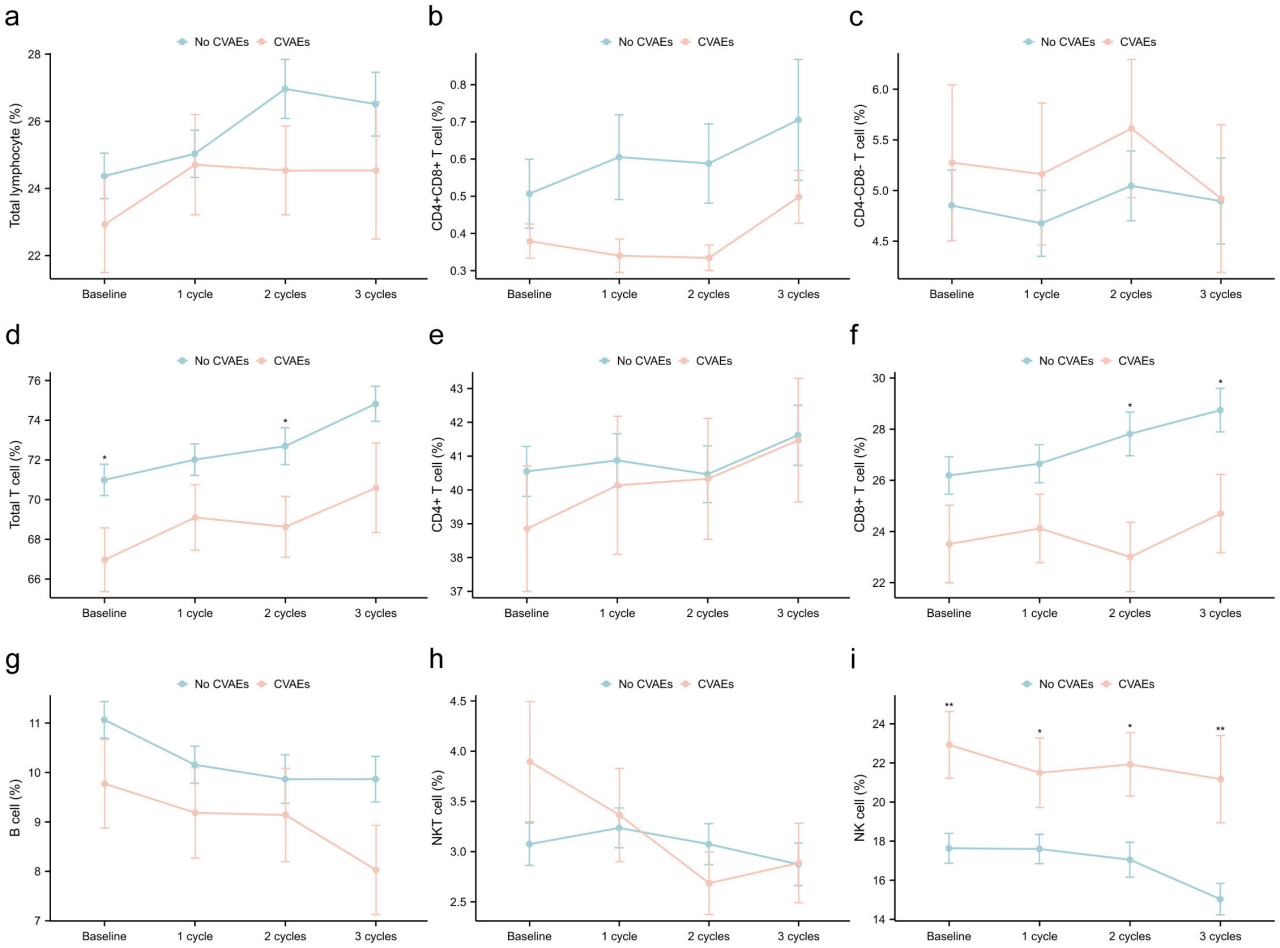
Dynamics of immune cell subsets in patients with and without CVAEs at the baseline, first, second and third cycles treatment. **(a)** total lymphocyte/PBMC; **(b)** CD4+CD8+ T cell/lymphocyte; **(c)** CD4-CD8- T cell/lymphocyte; **(d)** total T cell/lymphocyte; **(e)** CD4+ T cell/lymphocyte; **(f)** CD8+ T cell/lymphocyte; **(g)** B cel/lymphocytel; **(h)** NKT cell/lymphocyte; **(i)** NK cell/lymphocyte. CVAEs, cardiovascular adverse events; PBMC, peripheral blood mononuclear cell. *p: <0.05; **p: <0.01; ***p: <0.001.

Furthermore, we investigated the dynamics of immune cell subsets in patients with and without CVAEs, and found significant differences in the distribution of immune cell subsets (**Figure 4**, **Supplementary Table S2**). Specifically, total T cell proportion exhibited differences at baseline (p0 = 0.037) and the first cycle of treatment (p1 = 0.047), while CD8+ T cell proportion showed marked differences at the second (p2 = 0.011) and third cycles (p3 = 0.041). Additionally, NK cell proportion demonstrated notable variations throughout the treatment process (p0 = 0.006, p1 = 0.038, p2 = 0.015, p3 = 0.003). The findings indicated that the dynamic changes in the proportion of total T cell, CD8+ T cell, and NK cell during ICI treatment were associated with CVAEs. Additionally, we observed that during ICI treatment, the proportion of CD8+ T cell and B cell was lower, while the proportion of NK cell was higher in the CVAEs group, consistent with the results from scRNA-seq analysis.

### 3.4 Subgroup analysis between ICI-associated myocarditis and other CVAEs

To rule out the potential influence of differences in the peripheral immune landscape between ICI-associated myocarditis and other CVAEs on our results, a subgroup analysis was conducted. The findings revealed no significant differences in immune cell subsets between the ICI-associated myocarditis group and the other CVAEs group (**Supplementary Table S3**), suggesting that both groups shared a similar peripheral immune landscape.

### 3.5 Factors influencing total T cell, CD8+ T cell and NK cell

**Figure 5:**
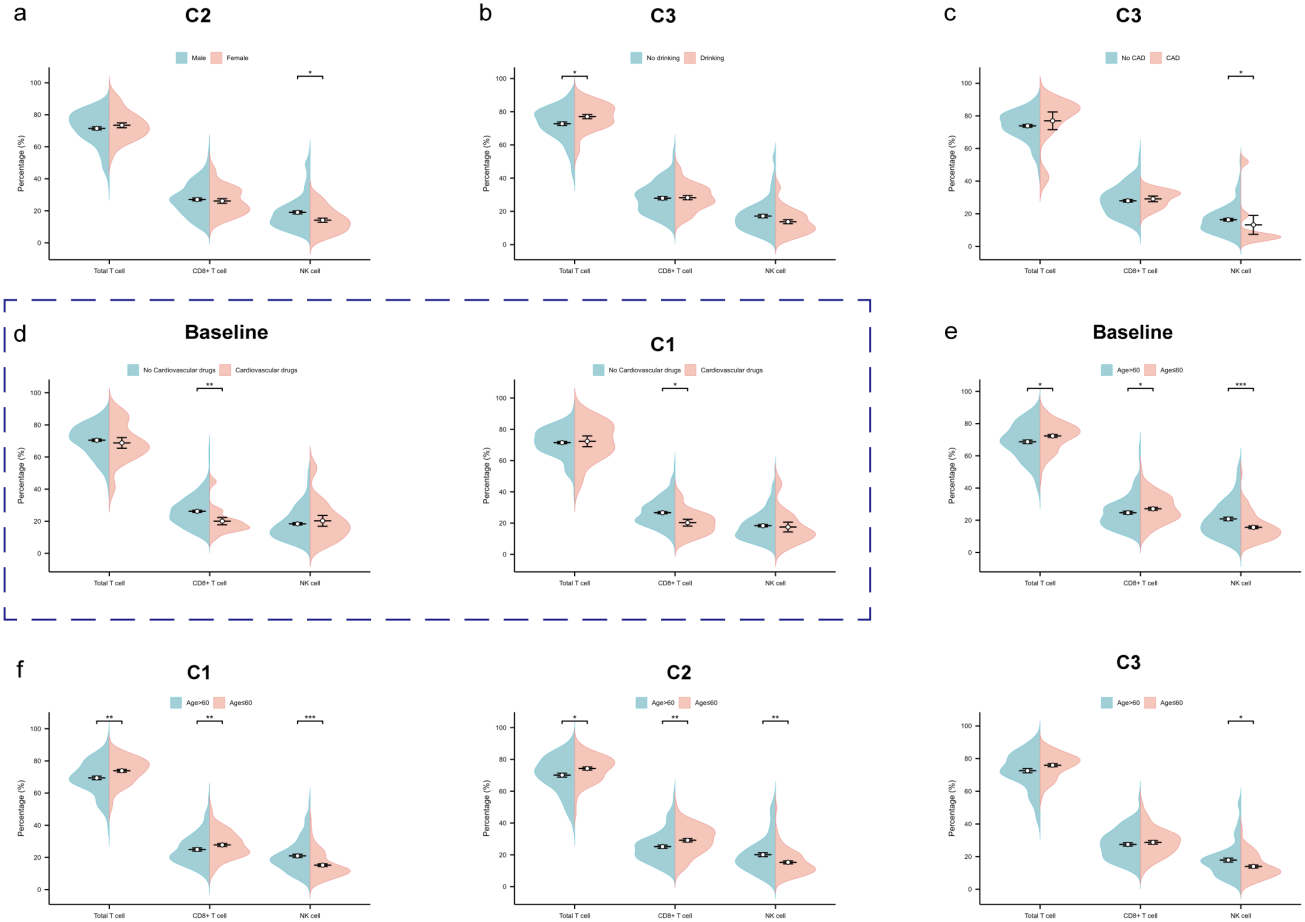
Factors influencing total T cell, CD8+ T cell and NK cell. **(a)** The beanplot of total T cell, CD8+ T cell and NK cell proportions by gender at the second treatment cycles. **(b)** The beanplot of total T cell, CD8+ T cell and NK cell proportions by alcohol drinking at the third treatment cycles. **(c)** The beanplot of total T cell, CD8+ T cell and NK cell proportions by coronary artery disease (CAD) at the third treatment cycles. **(d)** The beanplot of total T cell, CD8+ T cell and NK cell proportions by cardiovascular drugs at the baseline and third treatment cycles. **(e)** The beanplot of total T cell, CD8+ T cell and NK cell proportions by age at the baseline. **(f)** The beanplot of total T cell, CD8+ T cell and NK cell proportions by age at the first, second and third treatment cycles. C1: 1 cycle; C2: 2cycles; C3: 3cycles. *p: <0.05; **p: <0.01; ***p: <0.001.

We used the Wilcoxon rank-sum test to compare the total T cell, CD8+ T cell, and NK cell among subgroups at different time points (**Supplementary Table S4**). At the second treatment cycles, male patients expressed significantly higher NK cell compared to female patients (p2 = 0.020) (**Figure 5a**). At the third cycles, patients with a history of alcohol consumption showed a significant increase in total T cell (p3 = 0.019) *(* **Figure 5b** *)*, while those without CAD had higher NK cell (p3 = 0.023) (**Figure 5c**). Patients using cardiovascular medications had lower CD8+ T cell levels at baseline (p0 = 0.005) and at the first treatment cycle (p1 = 0.017) (**Figure 5d** *)*. Notably, patients aged >60 years consistently demonstrated significantly lower total T cell (p0 = 0.015, p1 = 0.003, p2 = 0.027) and CD8+ T cell (p0 = 0.014, p1 = 0.008, p2 = 0.002) at baseline, the first and second treatment cycles, but higher NK cell at all time points (p0<0.001, p1<0.001, p2 = 0.003, p3 = 0.034) *(* **Figures 5e, f**).

### 3.6 Risk factors of ICI-associated CVAEs

We employed logistic regression analysis to explore the risk factors associated with ICI-associated CVAEs. In univariate regression analysis, baseline total T cell (p=0.040) were identified as protective factors against CVAEs. Conversely, NK cell (p=0.008) were found to be risk factors for CVAEs. Further multivariate logistic regression analysis, after adjusting for potential confounders, revealed that only baseline NK cell proportion (p=0.009) were independently associated with an increased risk of ICI-associated CVAEs (**Table 2**).

**Table 2:** Univariate and multivariate logistic regression analysis with ICI-associated CVAEs.

Risk factors	Univariate analysis		Multivariable analysis	
	OR (95%CI)	P value	OR (95%CI)	P value
Demographic characteristics				
Age >60	1.778 (0.812-3.893)	0.150	1.541 (0.657-3.614)	0.320
Male	2.102 (0.667-6.102)	0.214	0.578 (0.184-1.816)	0.348
BMI, Kg/m2	1.077 (0.941-1.233)	0.284	1.079 (0.939-1.240)	0.283
Smoking (yes/no)	1.214 (0.574-2.567)	0.611	0.780 (0.316-1.929)	0.591
Alcohol drinking (yes/no)	1.125 (0.509-2.489)	0.771	1.146 (0.421-3.117)	0.789
Past medical history				
Coronary artery disease (yes/no)	2.717 (0.644-11.461)	0.174	2.076 (0.426-10.107)	0.366
Hypertension (yes/no)	1.897 (0.733-4.914)	0.187	1.421 (0.463-4.360)	0.539
Cardiovascular drugs (yes/no)	2.207 (0.648-7.514)	0.205	2.226 (0.614-8.070)	0.223
Baseline				
Total lymphocyte/PBMC (%)	0.981 (0.938-1.025)	0.384		
Total T cell/lymphocyte (%)	0.963 (0.929-0.998)	0.040	1.020 (0.925-1.124)	0.691
CD4+ T cell/lymphocyte (%)	0.982 (0.945-1.021)	0.362		
CD8+ T cell/lymphocyte (%)	0.967 (0.926-1.010)	0.134		
CD4+CD8+ T cell/lymphocyte(%)	0.663 (0.191-2.299)	0.517		
CD4-CD8- T cell/lymphocyte(%)	1.019 (0.944-1.100)	0.623		
B cell/lymphocyte(%)	0.940 (0.863-1.024)	0.159		
NK cell/lymphocyte (%)	1.048 (1.012-1.084)	0.008	1.047 (1.011-1.084)	0.009
NKT cell/lymphocyte (%)	1.089 (0.972-1.219)	0.141		

ICI, immune checkpoint inhibitor; CVAEs, cardiovascular adverse events.

### 3.7 Predictor of ICI-associated CVAEs

**Figure 6:**
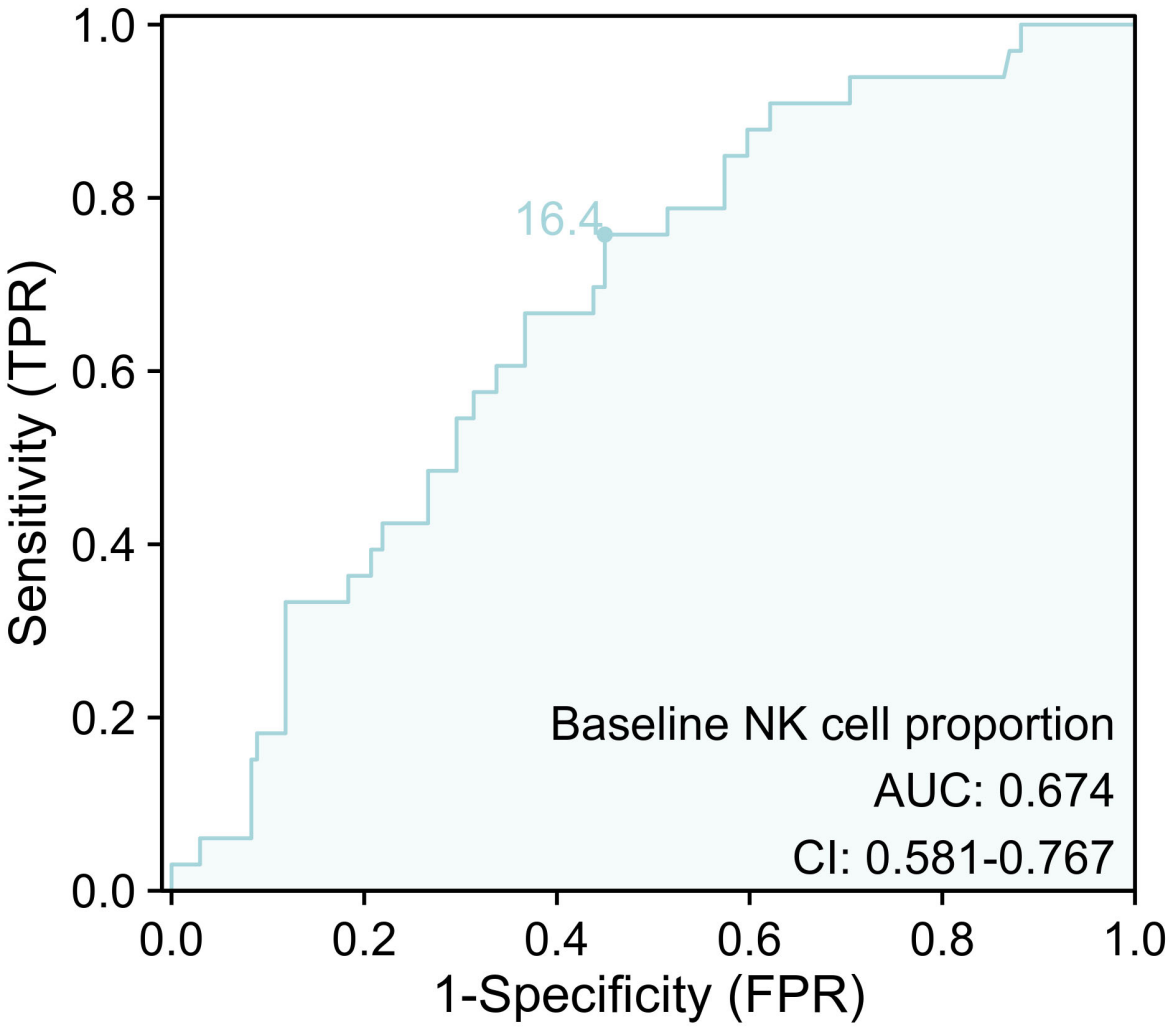
The ROC curve for baseline NK cell proportion for ICI-associated CVAEs. ICI, immune checkpoint inhibitor; CVAEs, cardiovascular adverse events.

**Figure 7:**
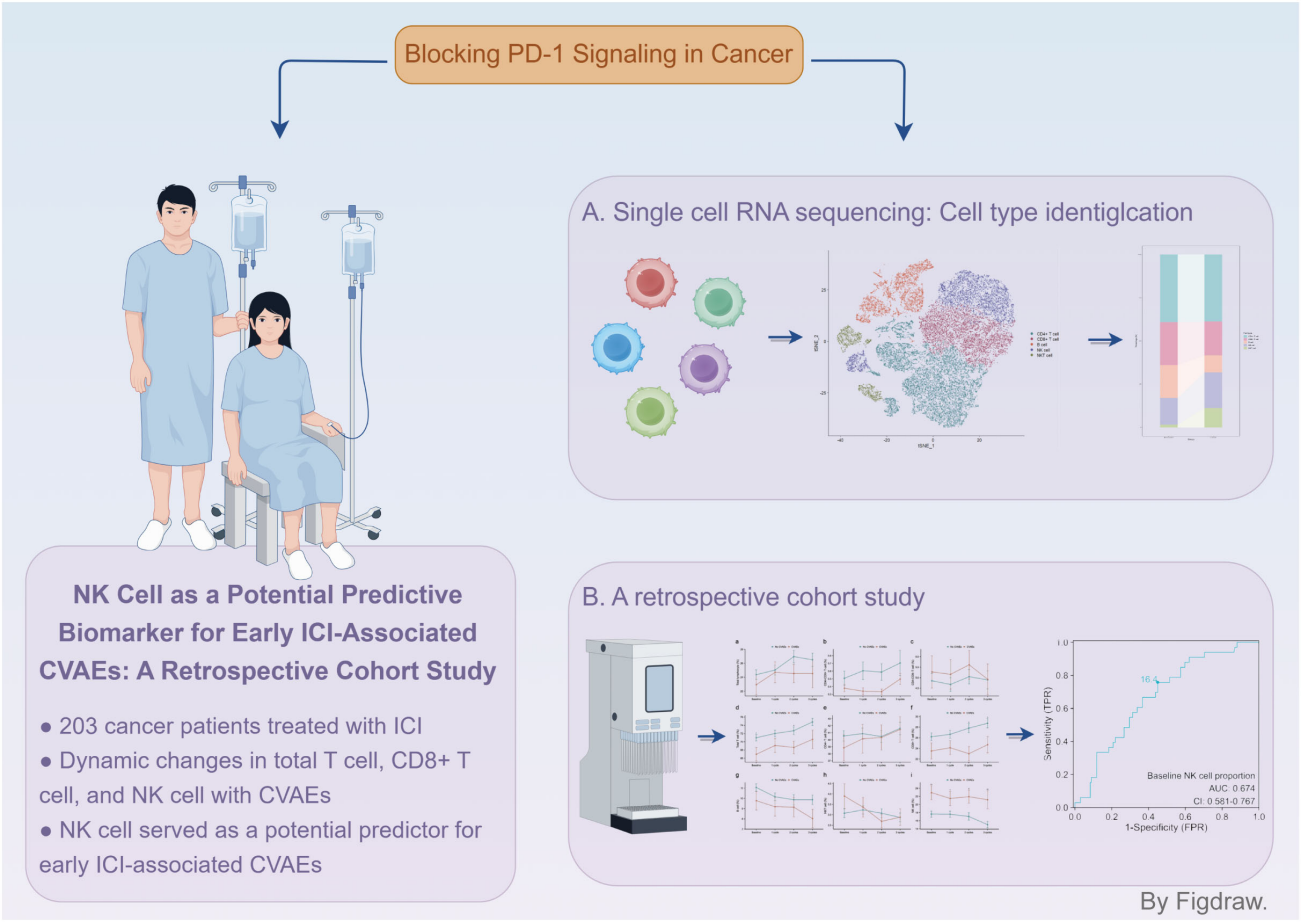
The baseline proportion of NK cell: a potential predictor for ICI-associated CVAEs.

The ROC curve was performed to investigate the predictive value of baseline NK cell proportion for ICI-associated CVAEs. The results revealed that the area under the curve (AUC) for baseline NK cell proportion was 0.674 (95% confidence interval [CI]: 0.581 to 0.767) (**Figure 6**). The optimal cutoff value for baseline NK cell proportion was determined to be 16.4%(**Figure 7**).

### 3.8 Effect of high NK cell on ICI-associated CVAEs after PSM

The PSM was employed to exclude the influence of confounding factors including age, gender, BMI, smoking status, alcohol consumption, coronary artery disease, hypertension, and cardiovascular medications on the final results. Based on the baseline NK cell proportion cutoff value, we categorized patients into high and low NK cell proportion groups. After PSM, we included confounding factors and matched 71 pairs of patients (**Supplementary Figure S3**). Chi-square tests were conducted, revealing that the differences between the two groups were eliminated (**Table 3**). Based on the matched cohort, nonparametric testing further confirmed that, after excluding confounding factors, the incidence of ICI-associated CVAEs was higher in the high baseline NK cell proportion group (**Table 3**).

**Table 3:** Characteristics between low and high baseline NK cell proportion group.

	Low baseline NK cell	High baseline NK cell	P-value
	(N=71)	(N=71)	
Group			
No CVAEs	67 (94.4%)	51 (71.8%)	<0.001
CVAEs	4 (5.6%)	20 (28.2%)	
Male	61 (85.9%)	63 (88.7%)	0.801
Age>60	40 (56.3%)	37 (52.1%)	0.736
BMI	23.1 (2.42)	22.9 (2.67)	0.694
Hypertension	6 (8.5%)	7 (9.9%)	1.000
CAD	1 (1.4%)	1 (1.4%)	1.000
Smoking	39 (54.9%)	41 (57.7%)	0.866
Alcohol drinking	25 (35.2%)	25 (35.2%)	1.000
Cardiovascular drugs	2 (2.8%)	5 (7.0%)	0.438

CVAEs, cardiovascular adverse events.

## 4 Discussion

Our study employed a two-step approach to identify immune cell subsets associated with ICI-associated CVAEs (**Figure 7**). First, we reanalyzed the scRNA-seq data from Han et al. to preliminarily investigate the changes in peripheral blood immune cells associated with ICI-associated CVAEs. Second, we validated the association between peripheral blood immune cell subsets and early ICI-associated CVAEs using a retrospective cohort study. Our findings ultimately suggested that the baseline NK cell proportion was a useful biomarker to predict early ICI-associated CVAEs. This result remained valid even after PSM analysis.\

ICIs, including programmed cell death protein 1 (PD-1) inhibitors, are monoclonal antibodies that block immune checkpoints (16). PD-1, induced in activated T cell, binds PD-L1/L2 to maintain tolerance but suppress anti-tumor immunity (17). PD-1-targeting ICIs block PD-1/PD-L1 signaling, relieving tumor cell inhibition of immune cell. In this study, we observed dynamic changes in peripheral blood immune cell subsets in patients undergoing ICI treatment. Specifically, total T cell exhibited an upward trend. Both CD4+ T cell and CD8+ T cell, the major subsets of T cell, also showed an increase during ICI treatment. This finding was consistent with prior studies (18–20), suggesting that T cell expansion likely followed ICI-induced immune activation. Subsequently, we further explored the correlation between immune cell subset changes and ICI-associated CVAEs. These results revealed significant differences in total T cell, CD8+ T cell, and NK cell between the two groups, suggesting their crucial role in early ICI-associated CVAEs, regardless of whether they were ICI-associated myocarditis or other CVAEs, as the subgroup analysis showed no differences. Both scRNA-seq results and clinical data analysis indicated that in patients with early ICI-associated CVAEs, the peripheral blood exhibited a reduced abundance of total T cell and CD8+ T cell, accompanied by an increased proportion of NK cell. Early histopathological examination of ICI-associated myocarditis demonstrated abundant infiltration of CD4+ and CD8+ T cell in myocardium, cardiac conduction system, and skeletal muscle (21). Therefore, expanded T cell may be recruited to tumor and myocardial tissues, promoting the occurrence of CVAEs, manifested as a decrease in peripheral blood T cell compared to patients without CVAEs.

NK cell, a small subset of lymphocytes, is integral to the innate immune response, playing a key role in immune surveillance and immune regulation. However, NK cell in the majority of cancer patients was dysfunctional (22). The tumor microenvironment created an inhibitory environment for NK cell, further promoting NK cell inactivation and weakening NK cell cytotoxicity (23). Importantly, while the correlation between NK cell quantity and cardiovascular disease (CVD) incidence remained controversial (24, 25), it was widely accepted that impaired NK cell function may be a contributing factor to CVD (26, 27). Consequently, the risk of CVD in cancer patients with impaired NK cell function was higher than in the general population.

In the present study, we observed that baseline NK cell proportion was an independent risk factor for early ICI-associated CVAEs. Higher levels of NK cell proportion in peripheral blood have been reported in patients with pembrolizumab-induced thyroiditis (28). Lou et al. (29) identified several key differential genes associated with ICI-associated myocarditis expressed in NK cell rather than T cell. NK cell play a regulatory role in the innate immunity and inflammation of ICI-associated myocarditis. Zhu et al. and Yu et al. independently observed a significant increase in the abundance of NK cell in the peripheral blood of patients with ICI-associated myocarditis (6, 7). However, neither study provided an extensive explanation for this finding. NK cell can enhance the anti-tumor effect of PD-1 inhibitors (30–32), and irAEs have been positively associated with ICI response (33). Based on these findings, NK cell may potentiate immune response while also contributing to excessive immune activation, resulting in myocardial tissue damage. Furthermore, Tsang et al. (34) have recently discovered that NK cell played a pivotal role in the rapid onset of myocarditis induced by mRNA COVID-19 vaccines. They have identified genetic risk profiles associated with KIR polymorphisms and NK cell-specific eQTLs. Although we could not provide a detailed description of the roles and biological functions of each NK cell subtype in ICI-associated CVAEs herein, it was noteworthy that several key mechanisms underlying ICI-associated CVAEs were closely linked to NK cell, warranting further attention.

Then, we used PSM to eliminate the impact of confounding factors on baseline NK cell proportion. After PSM, we found that patients with baseline NK cell proportion greater than 16.4% were at increased risk of CVAEs. Furthermore, the ROC curve indicated that baseline NK cell proportion had a predictive value for ICI-associated CVAEs. Given the heterogeneity of NK cell subsets between lung cancer and esophageal cancer (35), the predictive value of NK cell subpopulations for ICI-associated CVAEs requires further evaluation across different tumor types.

Our study has some limitations that should be mentioned. First, the results are subject to biases inherent to all retrospective studies. Second, in order to investigate the predictive value of immune cell subsets on early ICI-associated CVAEs, our study was focused on the first three cycles of ICI treatment in cancer patients; however, to determine whether these indices can predict late ICI-associated CVAEs and clinical outcomes such as ICI-associated CVAEs severity would require larger studies with longer follow-up. Furthermore, the limited sample size precluded stratified analysis of distinct immune cell subsets alterations across specific ICI-associated CVAEs subtypes, a critical gap that necessitates further investigation through larger, multicenter cohort studies.

A key strength of our study lies in our ability to identify critical immune cell subsets related to ICI-associated CVAEs by monitoring the dynamic changes in immune subsets proportions throughout immunotherapy. Additionally, considering the limitations of established cardiac biomarkers for ICI-associated CVAEs, our study evaluated the predictive value of immune cell subsets for early ICI-associated CVAEs. The baseline NK cell proportion emerged as an effective and potential predictive marker for early ICI-associated CVAEs. The specific cut-off value for the baseline NK cell proportion was determined using ROC analysis. Integrating NK cell proportion assessments into routine clinical practice could enhance risk stratification processes, ultimately informing treatment decisions and optimizing therapeutic strategies in oncology. Future studies are warranted to further elucidate the mechanisms by which NK cells contribute to CVAEs in patients undergoing ICI therapy. Understanding the underlying biological processes may lead to the development of targeted strategies aimed at modulating NK cell activity, thereby improving patient outcomes.

## 5 Conclusions

The baseline proportion of NK cell may serve as a potential predictor for early-onset ICI-associated CVAEs. Further prospective multicenter studies are warranted to validate the utility of NK cell in the assessment and risk stratification of early ICI-associated CVAEs.

## Data Availability

The original contributions presented in the study are included in the article/**Supplementary Material**. Further inquiries can be directed to the corresponding author.
