# Supplementary figures and images for: Natural killer cell as a potential predictive biomarker for early immune checkpoint inhibitor-associated cardiovascular adverse events: a retrospective cohort study
Source: Front Oncol. 2025 Jul 16;15:1556373. doi: 10.3389/fonc.2025.1556373 (PMC12307152; doi:10.3389/fonc.2025.1556373)

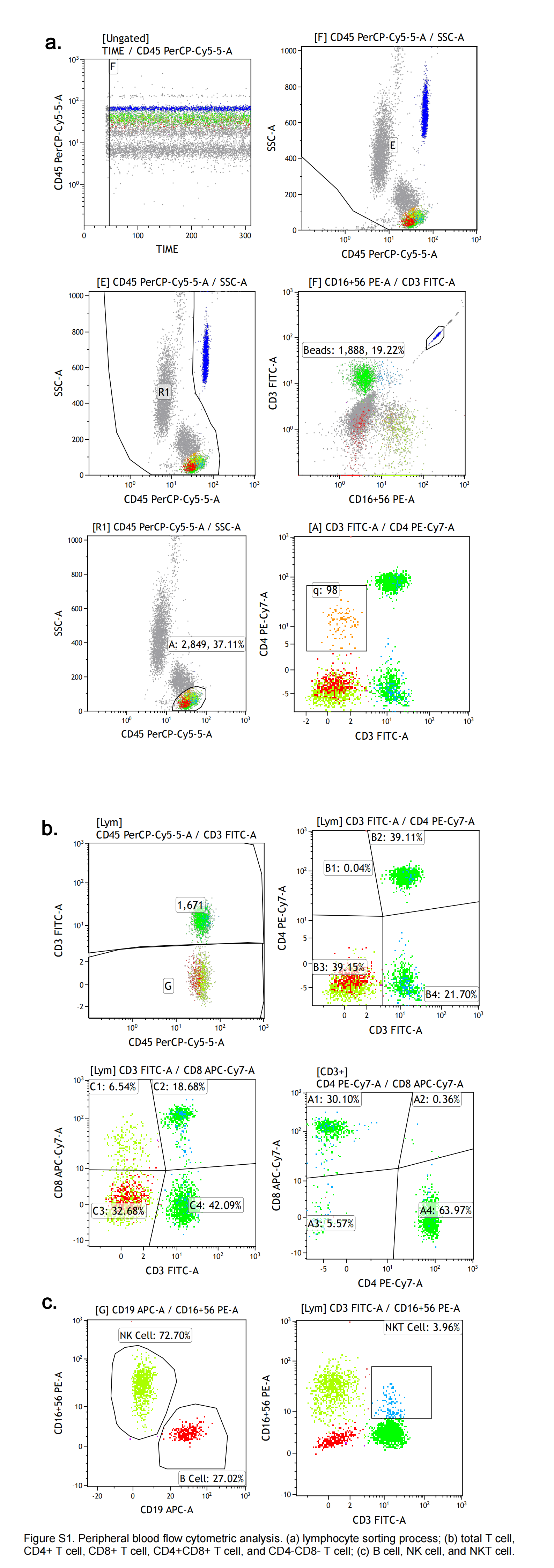

Supplement: Supplementary file 1 [file Image1.tif]

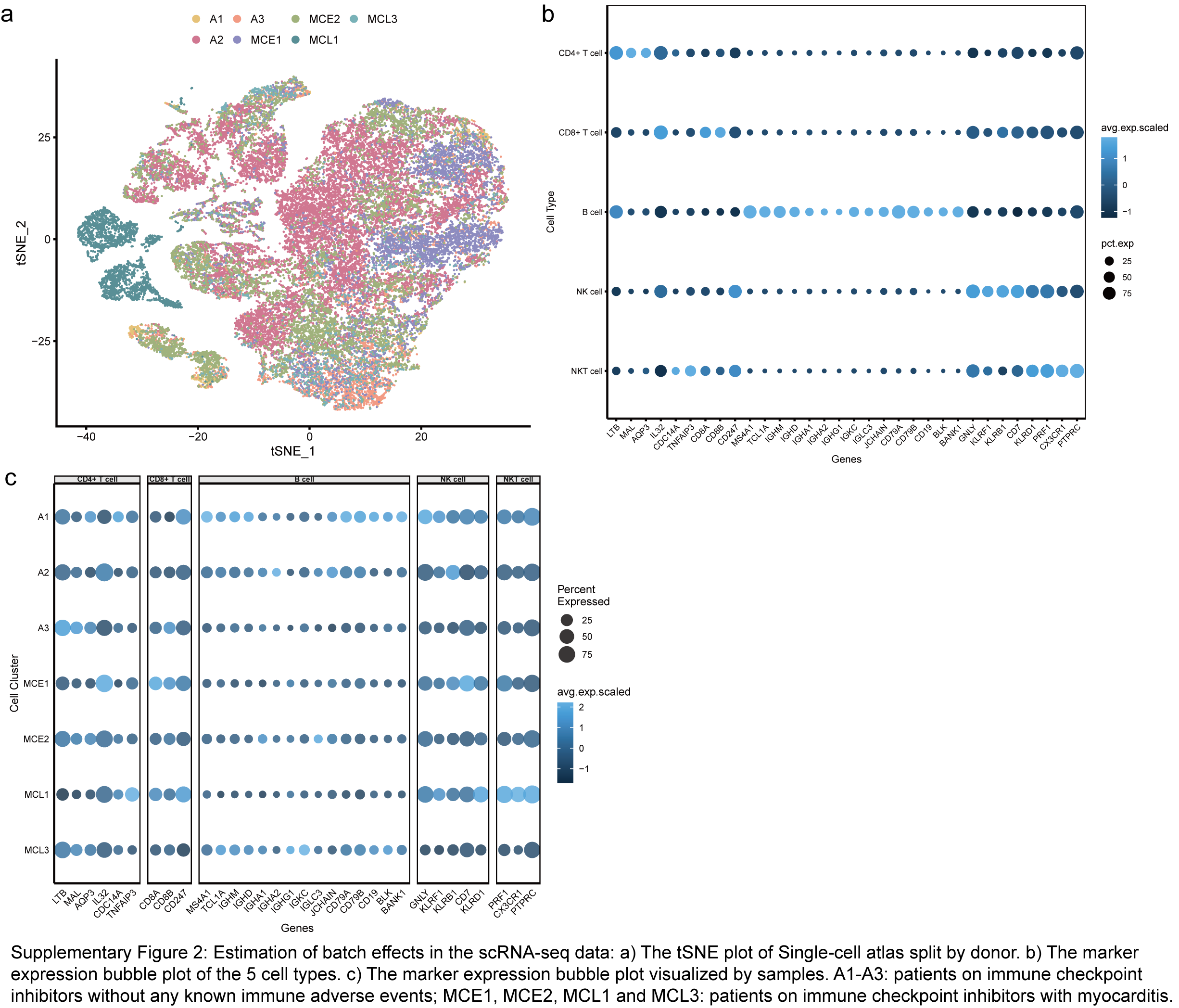

Supplement: Supplementary file 2 [file Image2.tif]

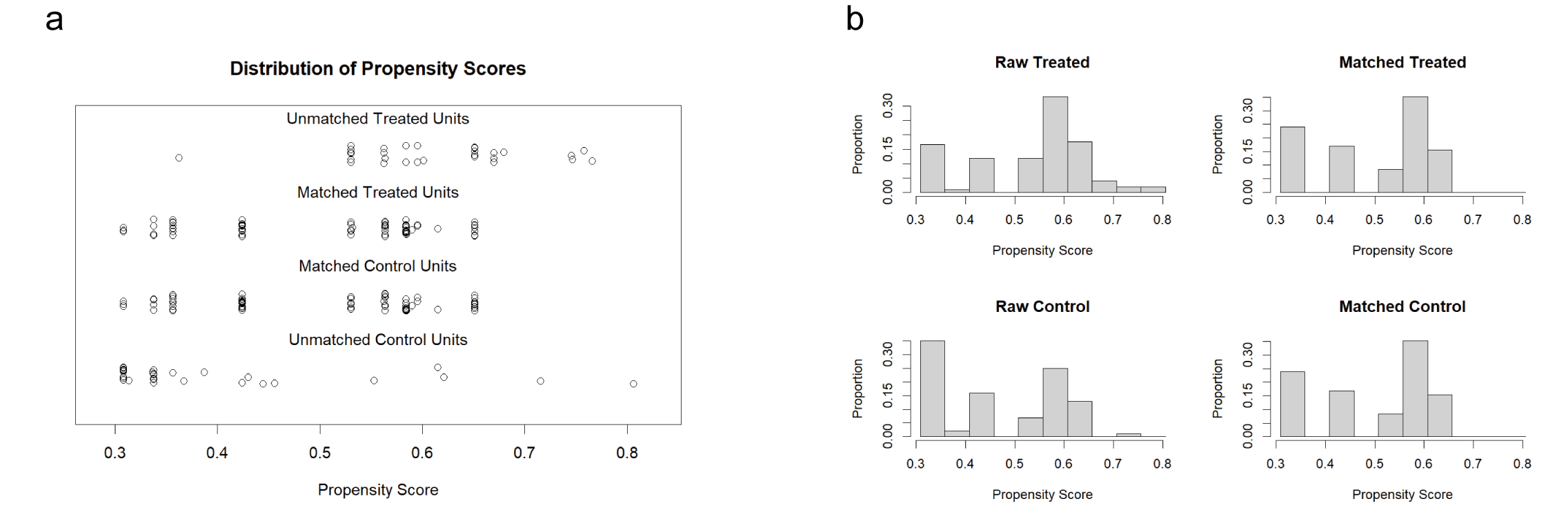

Supplement: Supplementary file 3 [file Image3.tif]
