# Supplementary material for: Natural killer cell as a potential predictive biomarker for early immune checkpoint inhibitor-associated cardiovascular adverse events: a retrospective cohort study
Source: Front Oncol. 2025 Jul 16;15:1556373. doi: 10.3389/fonc.2025.1556373 (PMC12307152; doi:10.3389/fonc.2025.1556373)
Supplement: Supplementary file 5 [file Table2.docx]

**Supplementary materials**

***Supplementary Table 2.*** Differences in immune cell subsets between patients with and without CVAEs at baseline, 1 cycle, 2 cycles, and 3 cycles. P-values were calculated using the Wilcoxon rank sum test.

|  | **Total lymphocyte/PBMC (%)** | **Total T cell/lymphocyte (%)** | **CD4+ T cell/lymphocyte (%)** | **CD8+ T cell/lymphocyte (%)** | **CD4+CD8+ T cell/lymphocyte(%)** | **CD4-CD8- T cell/lymphocyte(%)** | **B cell/lymphocyte(%)** | **NK cell/lymphocyte (%)** | **NKT cell/lymphocyte (%)** |
| --- | --- | --- | --- | --- | --- | --- | --- | --- | --- |
| **Baseline** | 0.386 | 0.037 | 0.364 | 0.134 | 0.547 | 0.624 | 0.158 | 0.006 | 0.133 |
| **1 cycle** | 0.849 | 0.133 | 0.710 | 0.156 | 0.304 | 0.541 | 0.295 | 0.038 | 0.794 |
| **2 cycles** | 0.204 | 0.047 | 0.944 | 0.011 | 0.245 | 0.467 | 0.508 | 0.015 | 0.381 |
| **3 cycles** | 0.378 | 0.051 | 0.944 | 0.041 | 0.552 | 0.980 | 0.084 | 0.003 | 0.978 |

***Supplementary Table 3.*** Immune cell subsets in the peripheral blood between ICI-associated myocarditis and other CVAEs.

|  | **Myocarditis (N=5)** | **Other CVAEs (N=28)** | **P value** |
| --- | --- | --- | --- |
| **Baseline** |  |  |  |
| Total lymphocyte/PBMC (%) | 20.834±6.503 | 23.304±8.536 | 0.561 |
| Total T cell/lymphocyte (%) | 71.302±11.387 | 66.195±8.797 | 0.298 |
| CD4+ T cell/lymphocyte (%) | 36.680±2.599 | 39.241±11.513 | 0.591 |
| CD8+ T cell/lymphocyte (%) | 32.028±9.370 | 21.988±7.820 | 0.027 |
| CD4+CD8+ T cell/lymphocyte(%) | 0.506±0.416 | 0.356±0.232 | 0.782 |
| CD4-CD8- T cell/lymphocyte(%) | 3.322±1.231 | 5.623±4.700 | 0.295 |
| B cell/lymphocyte(%) | 10.220±4.911 | 9.693±5.271 | 0.821 |
| NK cell/lymphocyte (%) | 18.092±8.286 | 23.784±9.937 | 0.238 |
| NKT cell/lymphocyte (%) | 6.084±5.851 | 3.506±2.808 | 0.064 |
| **1 cycle** |  |  |  |
| Total lymphocyte/PBMC (%) | 18.240±6.221 | 25.999±8.003 | 0.064 |
| Total T cell/lymphocyte (%) | 67.830±11.571 | 69.354±8.748 | 0.748 |
| CD4+ T cell/lymphocyte (%) | 34.874±4.400 | 41.190±11.876 | 0.193 |
| CD8+ T cell/lymphocyte (%) | 29.732±6.770 | 22.996±7.044 | 0.123 |
| CD4+CD8+ T cell/lymphocyte(%) | 0.382±0.300 | 0.332±0.240 | 0.937 |
| CD4-CD8- T cell/lymphocyte(%) | 3.830±1.681 | 5.428±4.114 | 0.413 |
| B cell/lymphocyte(%) | 10.184±3.738 | 8.985±5.274 | 0.597 |
| NK cell/lymphocyte (%) | 21.700±9.700 | 21.454±9.942 | 0.957 |
| NKT cell/lymphocyte (%) | 5.084±0.569 | 3.020±2.648 | 0.085 |
| **2 cycles** |  |  |  |
| Total lymphocyte/PBMC (%) | 22.865±2.244 | 24.772±7.945 | 0.713 |
| Total T cell/lymphocyte (%) | 73.178±11.352 | 67.980±8.282 | 0.348 |
| CD4+ T cell/lymphocyte (%) | 38.450±4.127 | 40.598±10.738 | 0.681 |
| CD8+ T cell/lymphocyte (%) | 30.738±6.952 | 21.900±7.213 | 0.080 |
| CD4+CD8+ T cell/lymphocyte(%) | 0.455±0.268 | 0.317±0.181 | 0.816 |
| CD4-CD8- T cell/lymphocyte(%) | 4.873±2.257 | 5.718±4.056 | 0.689 |
| B cell/lymphocyte(%) | 9.268±5.719 | 9.121±5.379 | 0.961 |
| NK cell/lymphocyte (%) | 17.343±6.974 | 22.583±9.345 | 0.331 |
| NKT cell/lymphocyte (%) | 3.860±1.285 | 2.518±1.781 | 0.264 |
| **3 cycles** |  |  |  |
| Total lymphocyte/PBMC (%) | 20.293±8.353 | 25.384±10.272 | 0.350 |
| Total T cell/lymphocyte (%) | 70.263±13.064 | 70.660±10.966 | 0.940 |
| CD4+ T cell/lymphocyte (%) | 36.190±4.848 | 42.526±9.305 | 0.212 |
| CD8+ T cell/lymphocyte (%) | 30.465±7.795 | 23.549±7.080 | 0.146 |
| CD4+CD8+ T cell/lymphocyte(%) | 0.685±0.418 | 0.461±0.331 | 0.792 |
| CD4-CD8- T cell/lymphocyte(%) | 4.595±2.836 | 4.985±3.761 | 0.869 |
| B cell/lymphocyte(%) | 8.938±5.782 | 7.846±4.260 | 0.671 |
| NK cell/lymphocyte (%) | 20.398±8.574 | 21.326±11.533 | 0.849 |
| NKT cell/lymphocyte (%) | 3.990±1.167 | 2.667±2.009 | 0.264 |

***Supplementary Table 4.*** Factors influencing Total T cell, CD8+ T cell and NK cell. P-values were calculated using the Wilcoxon rank sum test.

|  | **Age>60** | **Male** | **Smoking** | **Alcohol drinking** | **Coronary artery disease** | **Hypertension** | **Cardiovascular drugs** |
| --- | --- | --- | --- | --- | --- | --- | --- |
| **Baseline** |  |  |  |  |  |  |  |
| Total T cell/lymphocyte (%) | 69.37(15.84), p=0.015 | 71.55(13.50), p=0.633 | 71.56(12.89), p=0.226 | 71.06(14.50), P=0.5328 | 70.41(14.91), p=0.9276 | 70.67(14.02), p=0.729 | 67.80(13.35), p=0.521 |
| CD8+ T cell/lymphocyte (%) | 23.23(11.78), p=0.014 | 25.22(12.10), p=0.430 | 25.36(11.57), p=0.975 | 26.72(11.14), P=0.690 | 27.71(13.47), p=0.960 | 25.05(10.39), p=0.253 | 17.93(5.42), p=0.005 |
| NK cell/lymphocyte (%) | 18.95(15.55), p<0.001 | 16.78(12.54), p=0.164 | 16.27(11.11), p=0.851 | 16.35(13.26), P=0.784 | 17.97(20.00), p=0.844 | 17.25(11.95), p=0.368 | 16.81(11.50), p=0.682 |
| **1 cycle** |  |  |  |  |  |  |  |
| Total T cell/lymphocyte (%) | 69.29(13.39), p=0.003 | 72.83(12.74), p=0.384 | 72.87(12.38), p=0.301 | 72.16(14.22), p=0.8575 | 78.04(9.73), p=0.309 | 68.85(15.22), p=0.141 | 71.76(16.42), p=0.698 |
| CD8+ T cell/lymphocyte (%) | 22.55(12.00), p=0.008 | 25.78(11.25), p=0.153 | 26.34(10.33), p=0.617 | 27.75(10.57), p=0.449 | 29.45(6.59), p=0.938 | 23.36(10.90), p=0.136 | 20.08(9.36), p=0.017 |
| NK cell/lymphocyte (%) | 19.36(11.55), p<0.001 | 16.65(11.22), p=0.372 | 16.39(11.07), p=0.975 | 16.39(14.17), p=0.930 | 9.50(10.63), p=0.160 | 23.69(16.42), p=0.079 | 13.87(10.65), p=0.624 |
| **2 cycles** |  |  |  |  |  |  |  |
| Total T cell/lymphocyte (%) | 72.24(14.51), p=0.027 | 73.21(14.69), p=0.752 | 74.15(14.12), p=0.438 | 75.51(15.55), p=0.144 | 69.09(22.68), p=0.578 | 73.34(13.32), p=0.829 | 70.49(13.28), p=0.532 |
| CD8+ T cell/lymphocyte (%) | 23.76(11.84), p=0.002 | 27.18(14.54), p=0.645 | 27.00(11.61), p=0.829 | 27.50(9.74), p=0.709 | 26.74(12.77), p=0.429 | 26.48(7.72), p=0.453 | 35.65(21.71), p=0.075 |
| NK cell/lymphocyte (%) | 17.91(13.04), p=0.003 | 17.24(11.38), p=0.020 | 16.46(10.62), p=0.561 | 14.48(12.05), p=0.421 | 18.38(27.40), p=0.653 | 17.88(13.33), p=0.390 | 20.64(11.75), p=0.254 |
| **3 cycles** |  |  |  |  |  |  |  |
| Total T cell/lymphocyte (%) | 72.59(13.76), p=0.098 | 75.12(11.58), p=0.787 | 74.70(11.41), p=0.780 | 76.70(11.27), p=0.019 | 82.63(11.14), p=0.099 | 75.77(8.61), p=0.985 | 75.99(8.13), p=0.258 |
| CD8+ T cell/lymphocyte (%) | 27.19(13.27), p=0.360 | 27.83(11.35), p=0.422 | 27.62(9.12), p=0.572 | 27.17(9.89), p=0.720 | 30.61(7.30), p=0.539 | 26.96(9.58), p=0.677 | 26.77(7.77), p=0.883 |
| NK cell/lymphocyte (%) | 16.73(10.73), p=0.034 | 15.22(10.61), p=0.497 | 16.27(10.86), p=0.702 | 11.73(9.27), p=0.052 | 6.37(4.76), p=0.023 | 15.86(12.21), p=0.566 | 12.82(6.88), p=0.640 |
